# Supplementary material for: Stability follows efficiency based on the analysis of a large perovskite solar cells ageing dataset
Source: Nat Commun. 2023 Aug 12;14:4869. doi: 10.1038/s41467-023-40585-3 (PMC10423264; doi:10.1038/s41467-023-40585-3)
Supplement: Supplementary file 1 — Supplementary Information [file 41467_2023_40585_MOESM1_ESM.pdf]

Supplementary Information

Stability Follows Efficiency Based on the Analysis of a Large Perovskite Solar Cells Ageing Dataset

Noor Titan Putri Hartono[1,§]*, Hans Köbler[1,§], Paolo Graniero[2,3], Mark Khenkin[2], Rutger Schlatmann[2], Carolin Ulbrich[2], Antonio Abate[1]*

[1]Department Novel Materials and Interfaces for Photovoltaic Solar Cells, Helmholtz-Zentrum-Berlin, 12489 Berlin, Germany
[2]PVcomB, Helmholtz-Zentrum Berlin für Materialien und Energie, Helmholtz-Zentrum-Berlin, 12489 Berlin, Germany
[3]Department of Business Informatics, Freie Universität Berlin, 14195 Berlin, Germany

[§] Authors contributed equally to this work
*Corresponding authors: titan.hartono@helmholtz-berlin.de, antonio.abate@helmholtz-berlin.de

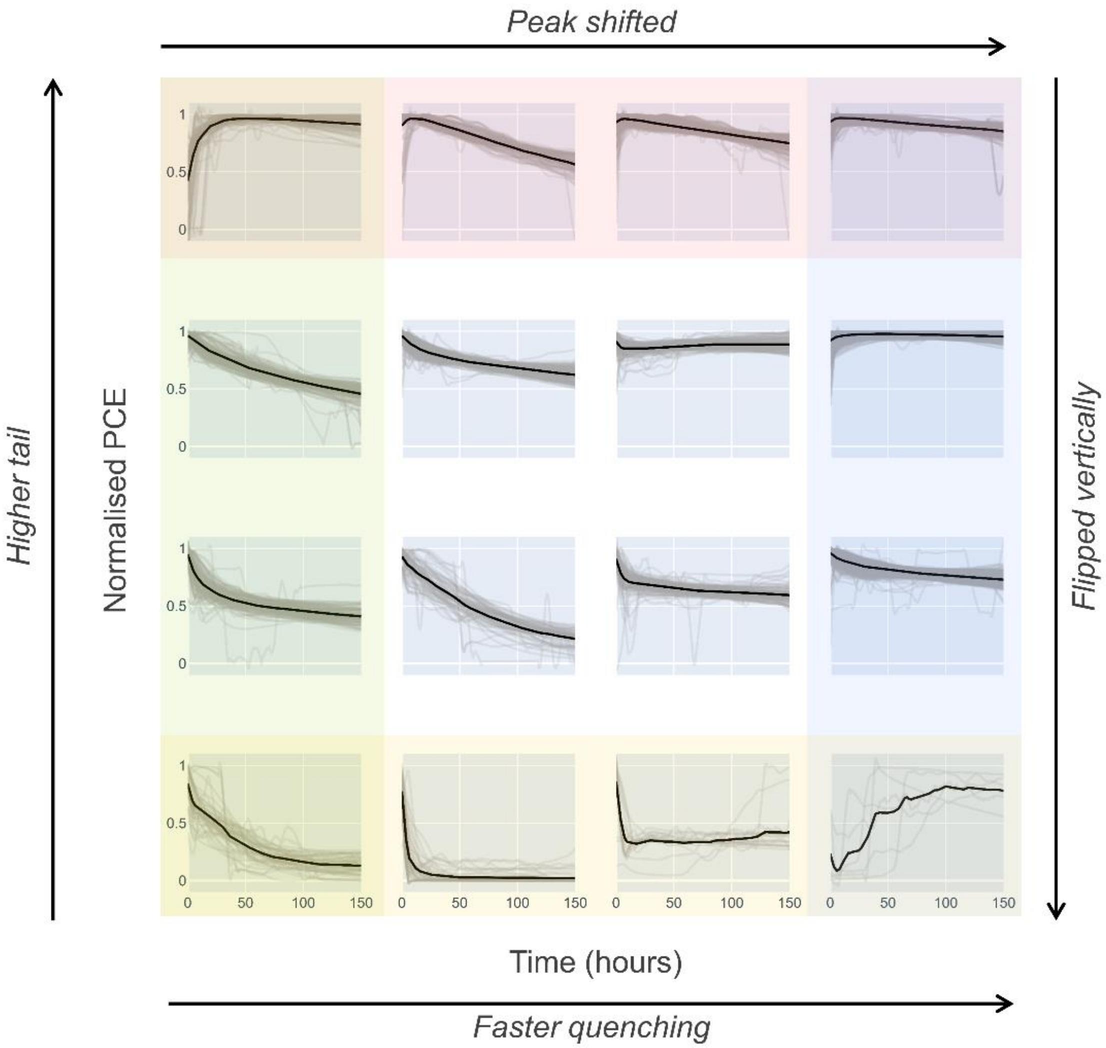

Supplementary Fig. 1. The overview of degradation curves within the dataset, based on MaxAbsScaler scikit-learn normalisation and SOM clustering with n = 16.

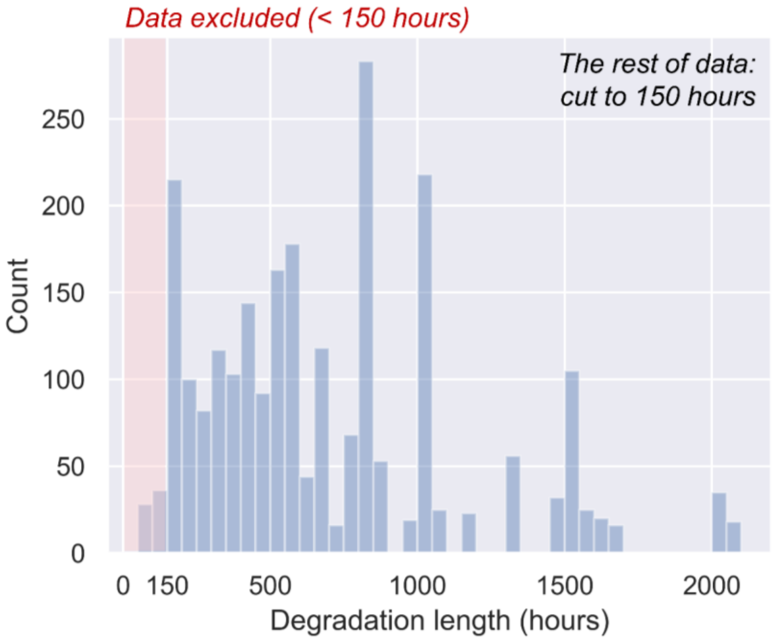

Supplementary Fig. 2. The distribution of degradation length in the dataset. The shaded red area indicates the data excluded in the analysis, which is shorter than 150 hours. Ageing tracks with a longer duration than 150 hours have been analysed in the first 150 hours only.

Detailed Dataset Description

The tested cells consist various electron transport layers (e.g. TiO_2-c, TiO_2-c/mesoporous TiO_2, C_{60}/BCP, LiF/C60/SnO_2, C_{60}/SnO_2, PCBM/BCP, PI/C_{60}, NaF/C_{60}/SnO_2, KF/C_{60}/SnO_2), hole transport materials (e.g. spiro-OMeTAD, HL-22, HL-31, PA02, PFI, PF, CSC5/spiro-OMeTAD, CSC5/TFI, CSC5/PF, SR2, SR3, SR5, SR1, SR4, TOPO/spiro-OMeTAD, MeO-2PACz, NiO, Me-4PACz, PEDOT:PSS, NiO Cu, NiO Zn, PTAA, NiO Mg, NiO/MeO-2PACz, NiO Cu/MeO-2PACz, 2PACz, PTAA/PFN), and top electrodes (e.g. Cu, Au, Ag, IZO). The complete list of device description is shown in **Supplementary Table 1**, and the degradation conditions are shown in **Supplementary Table 2**.

Bottom electrodes	• FTO: 502 cells<br>• ITO: 1,743 cells
Electron transport materials	*p-i-n*:<br>• C60/SnO_2: 621 cells<br>• C60/BCP: 406 cells<br>• LiF/C60/SnO_2: 383 cells<br>• PI/C60/SnO_2: 120 cells<br>• LiF/C60/BCP: 106 cells<br>• PCBM/BCP: 83 cells<br>• IPFC10/C60/SnO_2: 6 cells<br>• KF/C60/SnO_2: 5 cells

	• PIL/C60/SnO_2: 4 cells<br>• NaF/C60/SnO_2: 3 cells<br>• IPFC10/C60/BCP: 3 cells<br>• NaF/C60/BCP: 2 cells<br>• LiF/C60/0.1 wt% PEIE: 1 cell<br><br>*n-i-p*:<br>• TiO_2-c/TiO_2-mp: 187 cells<br>• TiO_2-c: 256 cells<br>• TiO_2-c/TiO_2-mp/LiTFSI: 36 cells<br>• V1036 MeO-2PACz: 23 cells
Absorbers	• 3CAT: 881 cells<br>• $CsPbI_3$: 218 cells<br>• FACs: 135 cells<br>• FAPI_Cl: 126 cells<br>• 3HAL: 103 cells<br>• ClCsMAFA: 85 cells<br>• $CsPbI_{1.8}Br_{1.2}$: 83 cells<br>• $MAFAPbI_3$: 61 cells<br>• FACsPI: 56 cells<br>• $FAPbI_3$: 56 cells<br>• MAPI: 50 cells<br>• $KCsFAPbI_3$: 48 cells<br>• 3HAL (5% Cl): 75 cells<br>• $FAPbI_3$ (10 mol% MACl): 41 cells<br>• CsFABrI: 37 cells<br>• 3CAT-PVDF: 33 cells<br>• ClCsFA: 32 cells<br>• 3CAT FACl: 26 cells<br>• $CsPbI_2Br$: 20 cells<br>• CsMAFA: 20 cells<br>• 3CAT - OXG: 10 cells<br>• $CsPbI_{2.2}Br_{0.8}$: 10 cells<br>• FAMACs: 9 cells<br>• CsFA: 8 cells<br>• ClCsFABrI: 7 cells<br>• 3HAL_WBG: 7 cells<br>• 3CAT_WBG: 5 cells<br>• 3CAT_BAI: 3 cells
Hole transport materials	*p-i-n*:<br>• MeO-2PACz: 607 cells<br>• 2PACz: 501 cells<br>• NiO: 200 cells<br>• Me-4PACz: 105 cells<br>• PEDOT:PSS: 56 cells

	• PTAA: 68 cells<br>• MeO-4PACz: 40 cells<br>• EADR03: 35 cells<br>• EADR04: 27 cells<br>• NiO Cu: 36 cells<br>• NiO Cu/MeO-2PACz: 18 cells<br>• NiO Zn: 11 cells<br>• NiO/MeO-2PACz: 17 cells<br>• NiO Mg: 9 cells<br>• PTAA/PFN: 7 cells<br>• NiO 2 Cu: 6 cells<br><br>*n-i-p*:<br>• Spiro-OMeTAD: 300 cells<br>• TOPO/spiro-OMeTAD: 27 cells<br>• PFI: 31 cells<br>• C60/SnO_2: 23 cells<br>• PF: 19 cells<br>• PA02: 17 cells<br>• HL-22: 16 cells<br>• HL-31: 14 cells<br>• SR3: 10 cells<br>• SR4: 10 cells<br>• SR5: 9 cells<br>• SR1: 9 cells<br>• CSC5/PF: 3 cells<br>• SR2: 6 cells<br>• CSC5/TFI: 5 cells<br>• CSC5/spiro-OMeTAD: 3 cells
Top electrodes	• Cu: 1,214 cells<br>• Au: 479 cells<br>• Ag: 491 cells<br>• IZO: 40 cells<br>• IZO Ag grid: 21 cells
Encapsulation	• Unencapsulated: 2,220 cells<br>• Encapsulated: 25 cells
Area	• 0.06 cm^2: 7 cells<br>• 0.16 cm^2: 1,626 cells<br>• 0.18 cm^2: 612 cells

Supplementary Table 1. The material list for each device layer in the dataset.

Atmosphere	• N_2: 2,245 cells

Filter	• UV-filter: 475 cells<br>• None: 1,770 cells
Temperature	The device temperature ranges between 20.0 and 85 °C, with the majority of devices aged at 25 °C. See **Supplementary Fig. 19** for a distribution of the test temperature.
Irradiation	• 1000 W/m^2: 2,245 cells

Supplementary Table 2. The degradation condition summary for the dataset.

MPPT Behaviour

Supplementary Fig. 3 shows schematically the two main cases of MPPT-behaviour that are observed for perovskite solar cells. This section discusses how they can be analysed in terms of PCE loss and reference point with respect to the stability figure of merit *ΔPCE,rel* which is used in this work.

In **Supplementary Fig. 3a**, an initial gain type of curve is depicted for which the efficiency after 150 hours is smaller than the initial efficiency ($PCE_{150\ h}$ < $PCE_{initial}$). During the stabilisation phase, the efficiency rises until it reaches a maximum after which it starts to decrease again. If one wants to calculate the loss that the solar cell has experienced after a certain amount of ageing time (here 150 hours), one can reference the losses either to the initial efficiency or the maximum efficiency. If referenced to the initial efficiency, the observed loss *ΔPCE,initial* is smaller than the value of *ΔPCE,max* if referenced to the maximum efficiency. Hence, referencing the initial PCE would give a lower loss and make the cell appear more stable in the case of the initial gain curve which drops below initial efficiency.

In **Supplementary Fig. 3b**, an initial gain type of curve is depicted for which the efficiency rises to maximum and then decays gently, so that the efficiency after 150 hours is still larger than the initial efficiency ($PCE_{150\ h}$ > $PCE_{initial}$). If the performance loss is calculated for this type of curve, one can again reference the losses either to the initial efficiency or the maximum efficiency. If referenced to the maximum efficiency, the losses *ΔPCE,max* are small in this example. If however referenced to the initial efficiency, the loss *ΔPCE,initial* would be negative and should rather be called a gain.

For the initial loss type of curve as depicted in **Supplementary Fig. 3c**, the curve decays at first exponentially until the behaviour becomes linear. Here, the initial efficiency equals the maximum efficiency. If the performance losses are calculated with respect to the maximum efficiency, the calculation takes into account the losses which happen during the stabilisation phase, and *ΔPCE,max = ΔPCE,initial* is not exclusively taking the linear part into account as it was the case for initial gain types of curves. If the performance loss is calculated only with respect to the linear part, one must exclude the initial loss and the calculated performance loss *ΔPCE,stabilised* is smaller than *ΔPCE,max* which makes the cell appear more stable.

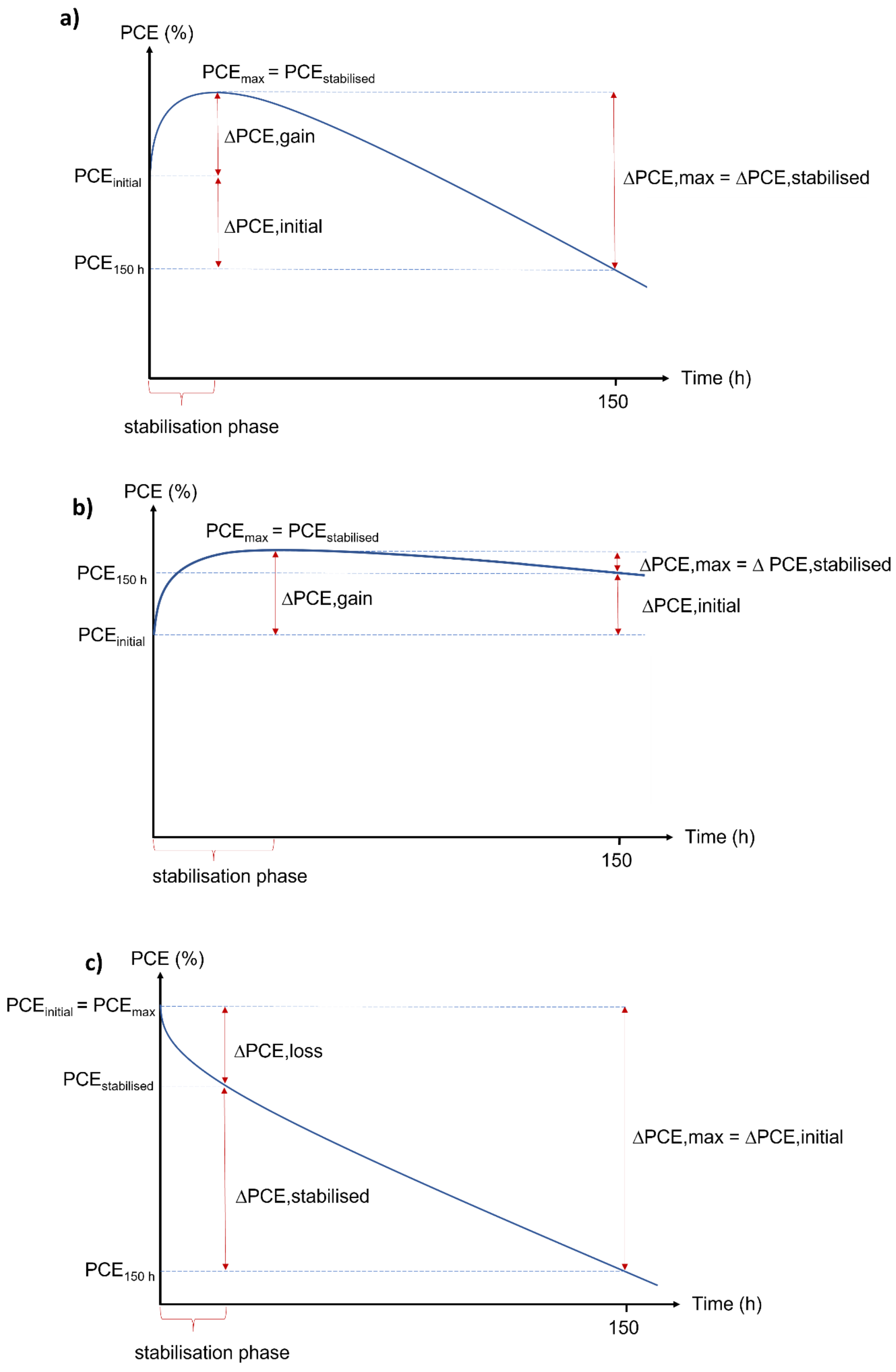

Supplementary Fig. 3. Schematics of different curve shapes. **a** initial gain type of curve where $PCE_{150\ h} < PCE_{initial}$, **b** initial gain type of curve where $PCE_{150\ h} > PCE_{initial}$, and **c** initial loss curve.

From the discussion it becomes clear that depending on the type of the ageing curve and depending on the choice of reference point, the value of performance losses can vary, thus over- or under-estimating the stability. and there is no fair way to compare the different curve shapes with the same reference point.

Yet, we chose to take *ΔPCE,max* as a universal figure of merit for all types of curves, and the reason is twofold.

First, if the calculation of losses is performed with respect to the maximum efficiency, the stabilisation phase is taken into account by calculating ΔPCE,max with respect to what has happened during the stabilisation phase for both the initial gain and the initial loss type of curve. First, if the calculation of losses is performed with respect to the maximum efficiency, for both the initial gain and the initial loss type of curve, the stabilisation phase is considered by calculating taken into account in the sense that *ΔPCE,max* is calculated with respect to what has happened during the stabilisation phase. For the initial loss curve type, this will increase the absolute value of the performance loss. However, if one looks at stability as the ability of the cell to maintain a high efficiency over time, this is justified since cells that have a strong initial loss are also less stable with respect to this definition of stability.

Secondly, one can read the maximum efficiency as the maximal capability of the system to produce energy. For initial loss types of cells, this potential is seen right at the beginning of the MPP-tracking, while for initial gain types of cells the maximum potential is reached after the stabilisation phase. Hence it appears to be a valid choice to reference the losses observed after a certain time of operation to the maximal capability of the solar cell.

Influence of band gaps

For the observed trend that high efficient devices also yield higher stability, we proposed the conservation of energy model, stating that in a lower efficient device less of the potentially available electric energy is extracted from the device and therefore dissipated in the device and available to cause degradation. However, with different band gaps, the theoretical maximum absorbable energy changes. Therefore, in some cases, a high efficient device with a band gap close to optimum might have even more energy left for dissipation and therefore degradation than a device with a less optimal band gap with low efficiency. For example, a device utilising $FAPbI_3$ with a band gap of 1.48 eV[1] with AM1.5G spectra,[2] can absorb ~542 W/m^2 of input power. The absorbed power of the solar cells is calculated in a simplified manner by integrating the AM1.5G solar spectrum for photon energies below the band gap, assuming the absorptance is a step function that equals one above band gap energy and zero otherwise while ignoring reflection and other losses. A $FAPbI_3$ device with 21% PCE would convert 210 W/m^2 absolute according to the definition of PCE (power conversion efficiency) shown in **Supplementary Eq. 1**, where P_{out} is the power extracted from the cell, and P_{in} is the power input.

$$\mathrm{PCE} = \frac{P_{\mathrm{out}}}{P_{\mathrm{in}}} \qquad \textbf{(Supplementary Eq. 1)}$$

The difference between the absorbed power and the actual converted power is the power that will be dissipated in the device and is potentially available to trigger degradation, and amounts to ~332 W/m^2 in the previous example. A $CsPbI_{1.8}Br_{1.2}$ wide-band gap perovskite with 1.93 eV can absorb less input irradiation than the $FAPbI_3$ perovskite due to its wider band gap (more photons of the spectrum are transmitted due to the wider band gap). The 1.93 eV $CsPbI_{1.8}Br_{1.2}$, perovskite can absorb ~334 W/m^2 of the input radiation and an 18% efficient device, which can convert 180 W/m^2 absolute power, will leave ~154 W/m^2 that can potentially trigger degradation. Hence, in this simplified calculation, the higher efficient $FAPbI_3$ device at 21% PCE has more power (~332 W/m^2) left in the device that can potentially trigger degradation than the $CsPbI_{1.8}Br_{1.2}$ device with lower efficiency of 18% (~154 W/m^2 left), which would contradict the statistical findings to be explained by the proposed model of energy conservation. The total power density absorbed by the solar cells across different band gaps calculated from the AM1.5G spectrum is shown in **Supplementary Fig. 4**.

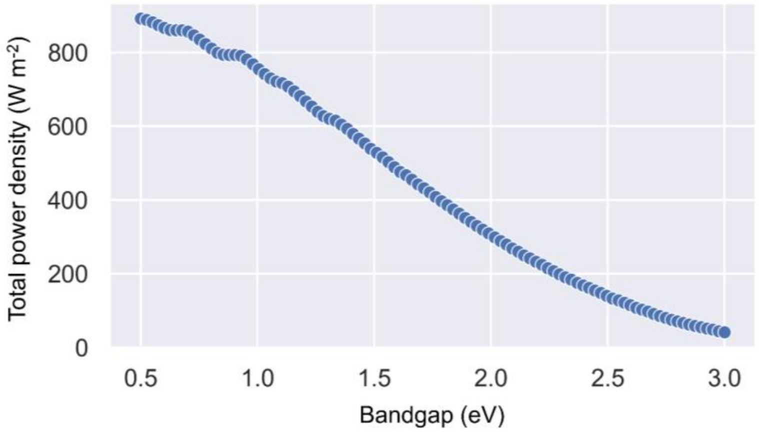

Supplementary Fig. 4. The total power density absorbed by the solar cells according to the AM1.5G spectrum.

To estimate the influence of band gaps on our statistical analysis, we compiled band gaps from literature for the most frequent occurring perovskites in our dataset as shown in **Supplementary Table 3.** Based on the band gaps of 1,597 cells (71.1% of the dataset), we calculated the theoretical maximum PCE according to the SQ-limit[3] and the absorbed power. We then calculated the potential power left in the device to trigger degradation for the efficiency of the maximum PCE group mean shown in **Fig. 2** and plotted it over the band gap in **Supplementary Fig. 5.**

Based on **Supplementary Fig. 5**, it is possible that a device with higher absolute PCE has more power left to be dissipated than a device with lower absolute PCE, which would contradict the explanation of our statistical finding with the conversion of energy model. Yet, this is only the case for materials with a significantly different band gap (thus, a significantly different absorbed power). An example is the comparison between the 1.93 eV $CsPbI_{1.8}Br_{1.2}$ cells in the 7.3% PCE group (dissipated power: 261 W/m^2) and the 1.73 eV $CsPbI_3$ cells in the 21.3% PCE group (dissipated power: 211 W/m^2). In these cases, the low efficiency device has less absorbed power than the device with high efficiency. We find this case to be uncommon and little in occurrence in our

database, indicated by its low frequency (< 5% of the data points) as seen in **Supplementary Fig. 5** Additionally, the majority of our dataset consists of 3CAT perovskite (881 data points) with the same or a very similar band gap. Therefore, we consider the influence of the band gap on our statistical analysis to be negligible.

**Absorbers**	**Number of cells**	**Band gap (eV)**	**Theoretical SQ limit PCE (%)**	**Theoretical absorbed power (W/m^2)**	**Reference(s)**
3CAT	881	1.64	30.2	466	Saliba, et al.[4]
$CsPbI_3$	218	1.73	28.6	424	Møller, C. K.[5]
FACs (referring to $Cs_{0.15}FA_{0.85}PbI_{2.55}Br_{0.45}$ from Emery, et al.[6])	135	~1.6	30.5	482	Tsarev, et al.[7]
3HAL	103	1.64-1.7	30.2-29.0	466-437	Chen, et al.[8]
$CsPbI_{1.8}Br_{1.2}$	83	1.93	24.4	334	Chen, et al.[9]
$FAPbI_3$	56	1.48	32.3	542	Eperon, et al.[1]
MAPI	48	1.57	31.1	498	Eperon, et al.[1]
3CAT-PVDF	33	1.64	30.2	466	Saliba, et al.[4]
$CsPbI_2Br$	20	1.92	24.6	338	Sutton, et al.[10]
3CAT - OXG	10	1.64	30.2	466	Saliba, et al.[4]
$CsPbI_{2.2}Br_{0.8}$	10	1.86	25.9	364	Yao, et al.[11]

Supplementary Table 3. The perovskite absorber materials in the dataset, with their theoretical Shockley-Queisser limit for efficiency and absorbed power. Band gaps were available for 1,597 cells (71.1% of the dataset).

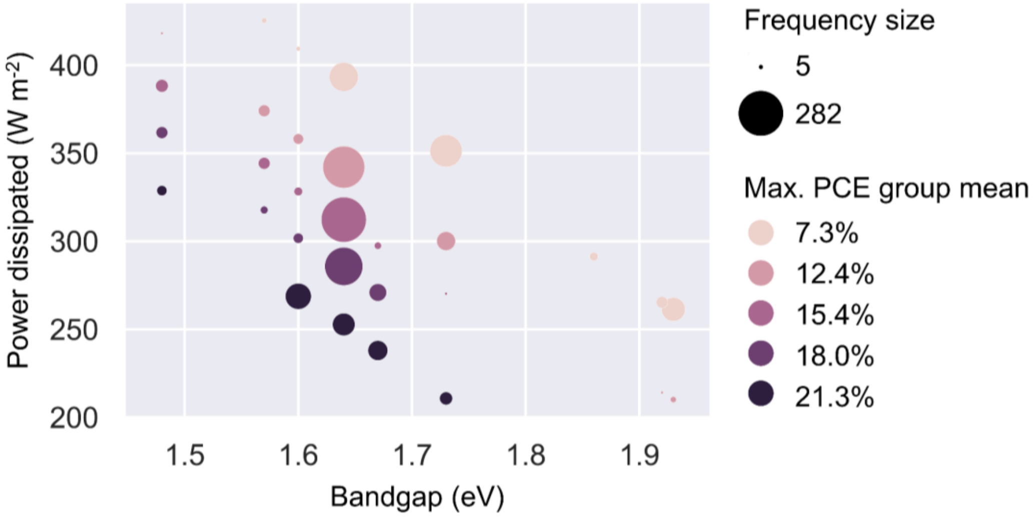

Supplementary Fig. 5. The calculated power dissipated and left in the device to potentially cause degradation for the 5 maximum PCE groups (mean: 7.3%, 12.4%, 15.4%, 18.0%, 21.3% for each group) in 71.1% of the dataset, based on the various power absorbed and bandgap. The size represents frequency.

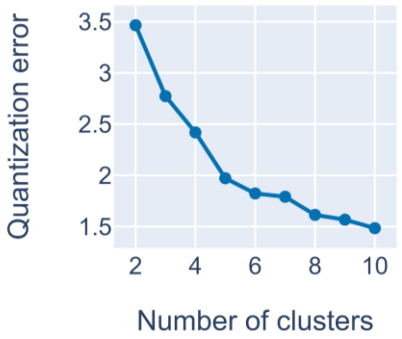

Supplementary Fig. 6. The quantisation error for SOM, showing n = 2-10. The optimum value is around n = 4.

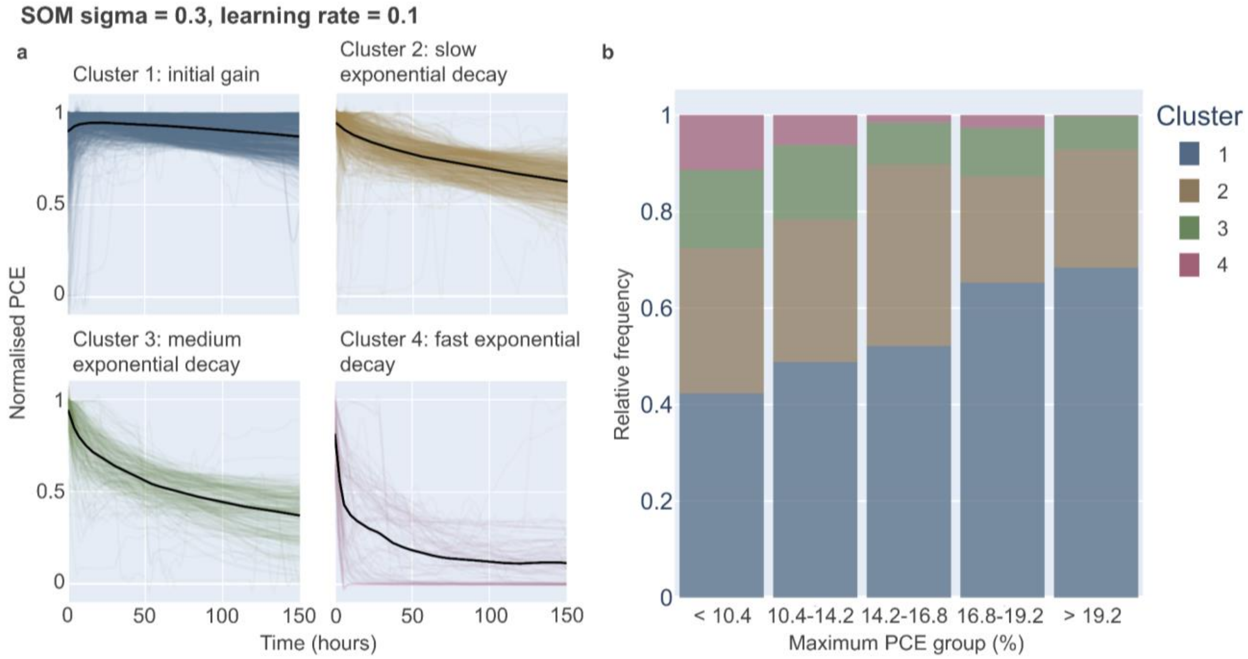

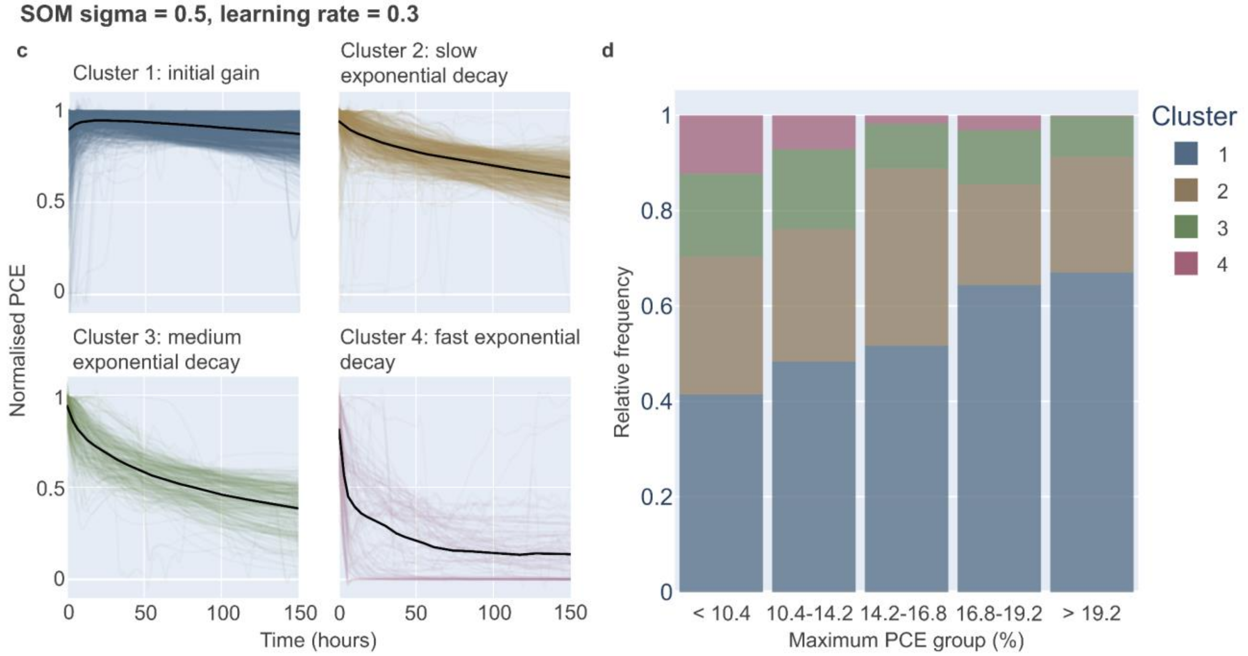

Supplementary Fig. 7: The self-organising map (SOM) for other hyperparameters. a The SOM for sigma = 0.3, learning rate = 0.1, and **b** the cluster distribution. **c** The SOM for sigma = 0.5, learning rate = 0.3, and **d** the cluster distribution. The figure shows that cluster 4, fast exponential decay, still decreases as we shift towards the higher maximum PCE group, with a small parameter change. On the other hand, cluster 1, initial gain increases as we shift towards the higher maximum PCE group, which is consistent with the previous findings.

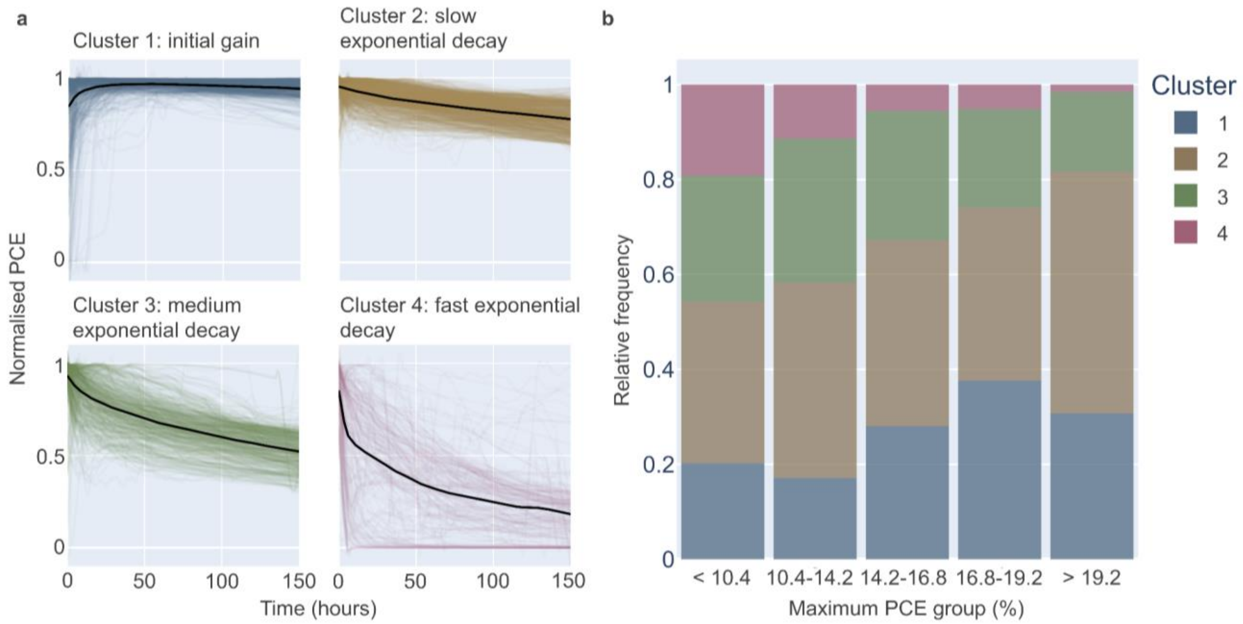

Supplementary Fig. 8: The *k*-means clustering results. a The *k*-means clustering results with DTW (dynamic time warping, with Barycenter averaging method), and **b** the cluster distribution. The figure shows cluster 4 fast exponential decay decreases as we shift towards the higher maximum PCE group.

Cluster	Max. PCE group (%)	Count	Mean	Median
Cluster 1, initial gain	< 10.4	190	13.8	13.1
	10.4-14.2	219	15.2	15.0
	14.2-16.8	234	12.5	8.8
	16.8-19.2	293	9.6	7.6
	> 19.2	307	12.9	13.0
Cluster 2, slow exponential decay	< 10.4	135	35.3	36.5
	10.4-14.2	133	37.0	36.1
	14.2-16.8	169	38.2	36.3
	16.8-19.2	99	37.6	37.2

	> 19.2	110	36.5	34.1
Cluster 3, medium exponential decay	< 10.4	73	64.9	63.6
	10.4-14.2	69	60.3	58.4
	14.2-16.8	39	64.4	61.9
	16.8-19.2	45	58.7	57.7
	> 19.2	31	59.0	53.8
Cluster 4, fast exponential decay	< 10.4	51	85.8	86.2
	10.4-14.2	28	93.1	99.9
	14.2-16.8	7	92.0	99.9
	16.8-19.2	12	82.7	81.9
	> 19.2	1	Not applicable	Not applicable

Supplementary Table 4. The mean and median for each cluster and max. PCE cluster (sigma = 0.5, learning rate = 0.1) based on **Fig. 4**.

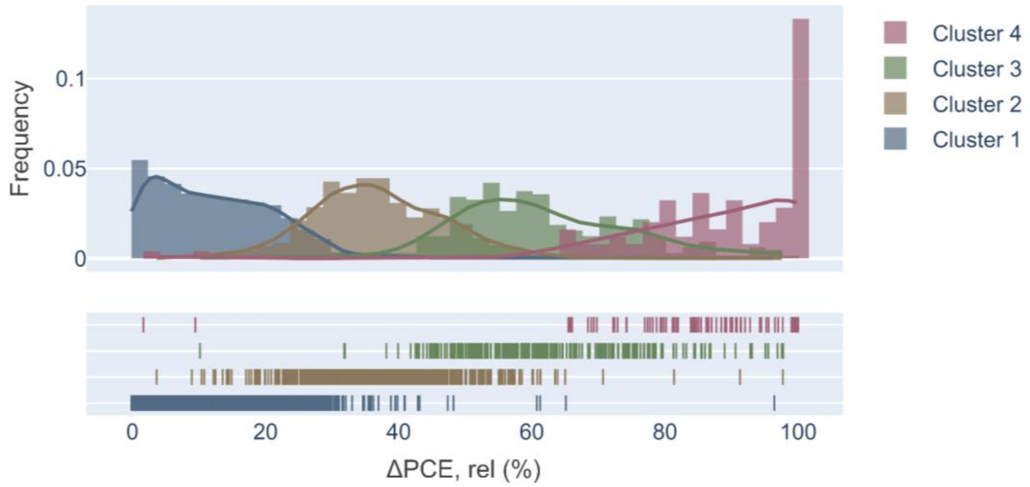

Supplementary Fig. 9: The distribution of *ΔPCE,rel* based on clusters. The histogram shows the frequency of *ΔPCE,rel*, and the density. It shows how each cluster is concentrated in a specific range of *ΔPCE,rel*).

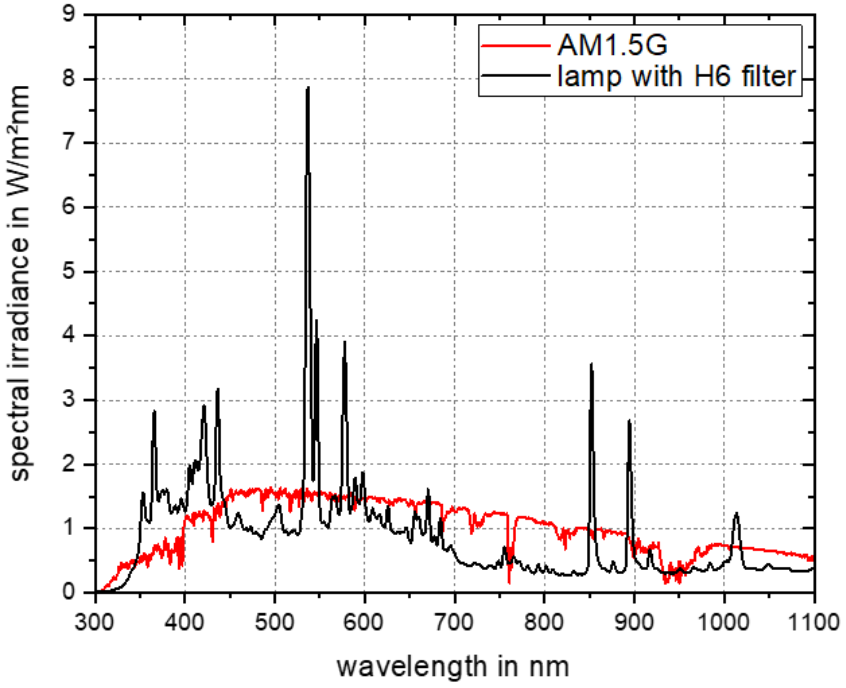

Supplementary Fig. 10. Spectrum of the lamp used to age solar cells in comparison to AM1.5G.

Degradation Time Length

We take a look at how long it takes to reach maximum PCE, and the result is shown in **Supplementary Fig. 11**. The majority of the dataset reaches maximum PCE before 150 hours (~96.8%, 2,245 ageing curves out of 2,320; the dataset only includes solar cells degraded under N_2 atmosphere).

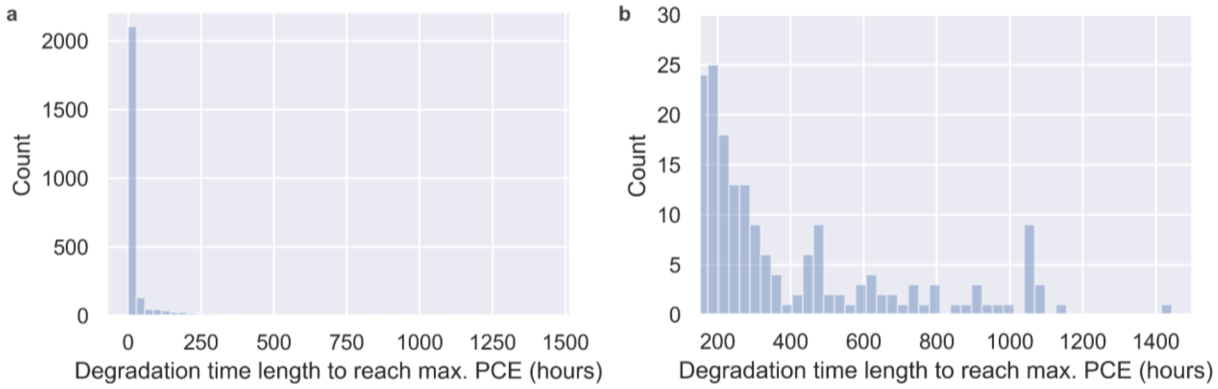

Supplementary Fig. 11. The histogram showing degradation time length taken to reach max. PCE; **a** shows the whole dataset, and **b** shows the inset of the histogram for time length between 150 and 1,500 hours.

We also performed the analysis for the dataset with cutoff time beyond 150 hours (300 and 500 hours), and observe that the regression fit still holds, shown in **Supplementary Fig. 12**. Note that the dataset is smaller in these cases (1,914 curves for 300 hours, and 1,497 for 500 hours) in comparison to 2,245 curves for 150 hours.

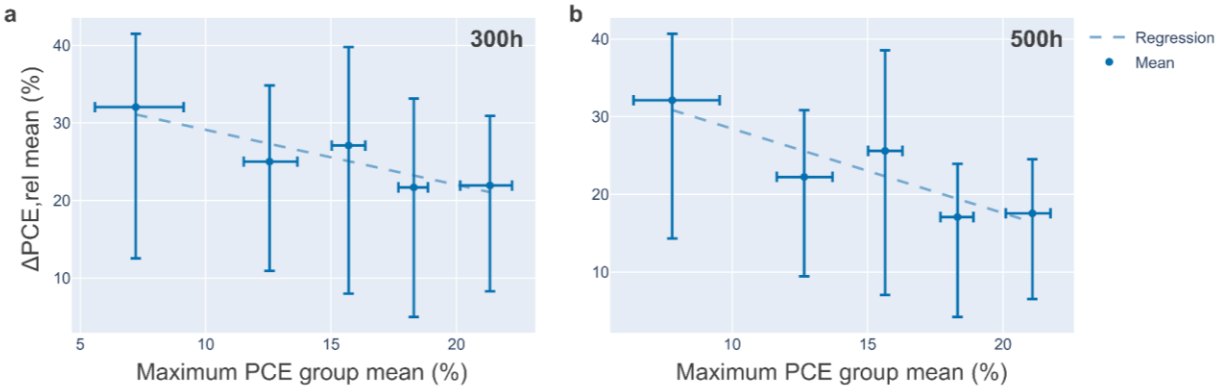

Supplementary Fig. 12: Regression fit of the maximum PCE group mean. The relationship between the mean for each maximum PCE group and its respective mean of the *ΔPCE,rel* is shown for ageing test time length of **a** 300 hours and **b** 500 hours. The dashed line is the regression fit of the mean points. The error bar for both axes represents the interquartile range between the 25th and 75th percentile of the maximum PCE group and the *ΔPCE,rel* data.

SOM Quantisation Error

Quantisation error is calculated as the following using the MiniSOM package.[12]

Quantisation error is the average Euclidean distance between each degradation curve x_i in the dataset containing n degradation curves, and the 'best-matching unit' weights vector $w_{I(x_i)}$, or the vector representing the 'main' shape of the corresponding cluster, shown in **Supplementary Eq. 2**.

$$\mathrm{QE} = \frac{1}{n}\sqrt{\sum_{i=1}^{n}(x_i - w_{I(x_i)})^2} \qquad \text{(Supplementary Eq. 2)}$$

The optimum cluster is defined as the minimum number of clusters with minimum quantisation error that captures the distinct characteristics or the 'main' shapes of the dataset. If the number of clusters is too high, the characteristics of the clusters start to overlap, and the clusters become indistinguishable. However, if the number of clusters is too low, the clusters are unable to capture the distinct characteristics of the dataset.

The standard way to look at the optimum cluster is by using an elbow plot, which plots the quantisation error against the number of clusters. The ideal elbow plot would look like **Supplementary Fig. 13**, which shows a significant drop of quantisation error at *n* = 3, and hence, is the optimum number of clusters for this particular example.

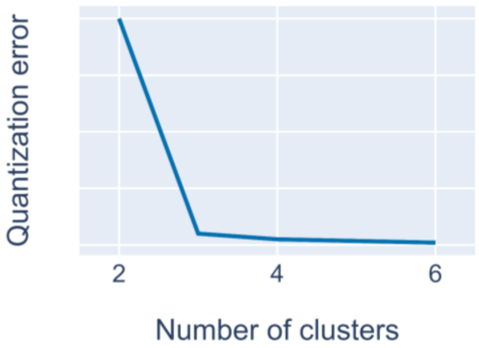

Supplementary Fig. 13. The ideal case of elbow plot, showing the quantisation error for various numbers of clusters. In this case, it is clear that the optimum number of clusters is n = 3.

The elbow plot calculated from our data in **Supplementary Fig. 6** also shows that the quantisation error decreases as the number of clusters increases. However, a steep drop of quantisation error is not observed here. The optimum number of clusters ranges between 4-6. This result is also supported by the result from k-nearest neighbour clustering (**Supplementary Fig. 14**).

When we performed SOM for the following number of clusters, n = 2, 5, and 6, as shown in **Supplementary Figs. 15, 16,** and **17**, respectively, we confirmed our selection of n = 4 clusters. In n = 2, cluster 2 (exponential decay) could clearly be broken down to more clusters. On the other hand, for n = 5, some clusters start overlapping/ becoming too similar, such as cluster 3 and 4. The same phenomenon is observed and is more pronounced for n = 6, such as cluster 1 and 4, and cluster 2 and 5.

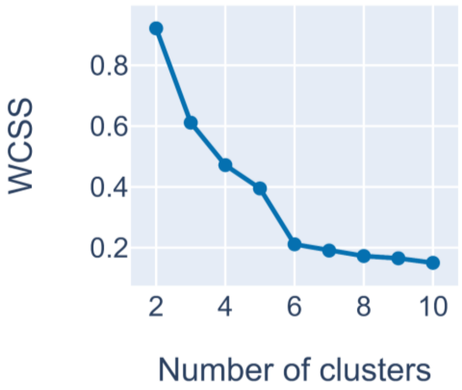

Supplementary Fig. 14: The elbow plot for *k*-means clustering. It shows the WCSS (within-cluster sum of squares), which is the sum of squared distance between each data point and the centroid in a cluster, for each k number of clusters.

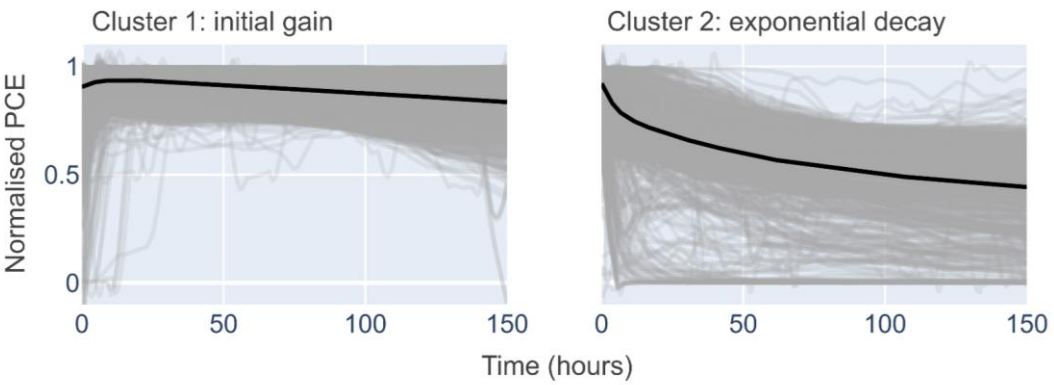

Supplementary Fig. 15. The SOM clusters for *n* = 2.

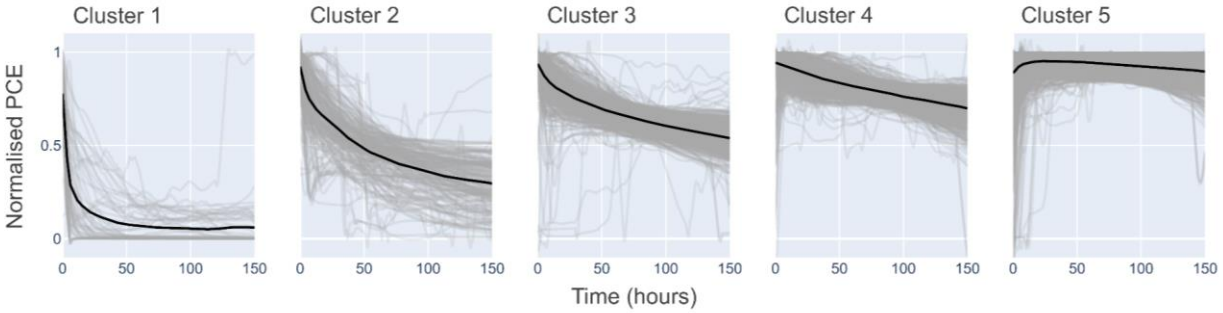

Supplementary Fig. 16. The SOM clusters for *n* = 5.

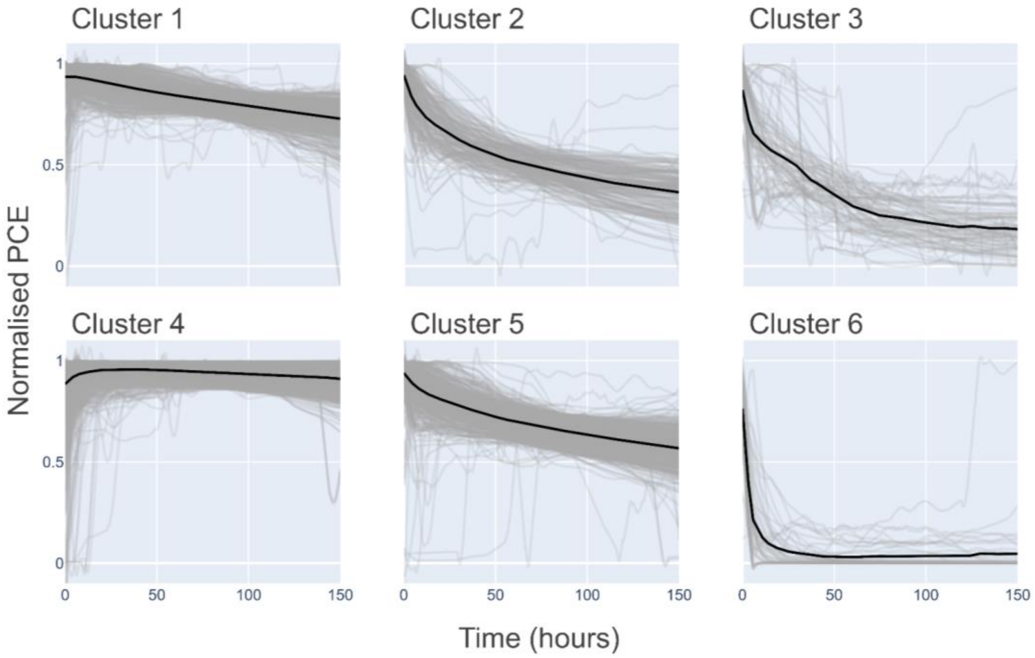

Supplementary Fig. 17. The SOM clusters for *n* = 6.

Device Architecture Impact on Clustering

The *n-i-p* devices (502 devices) are fewer than *p-i-n* devices (1,743 devices). There is a very small difference between the *p-i-n* and *n-i-p* degradation clusters, as shown in **Supplementary Fig. 18**. This implies that the general cluster shapes and conclusions drawn are independent of the device structure.

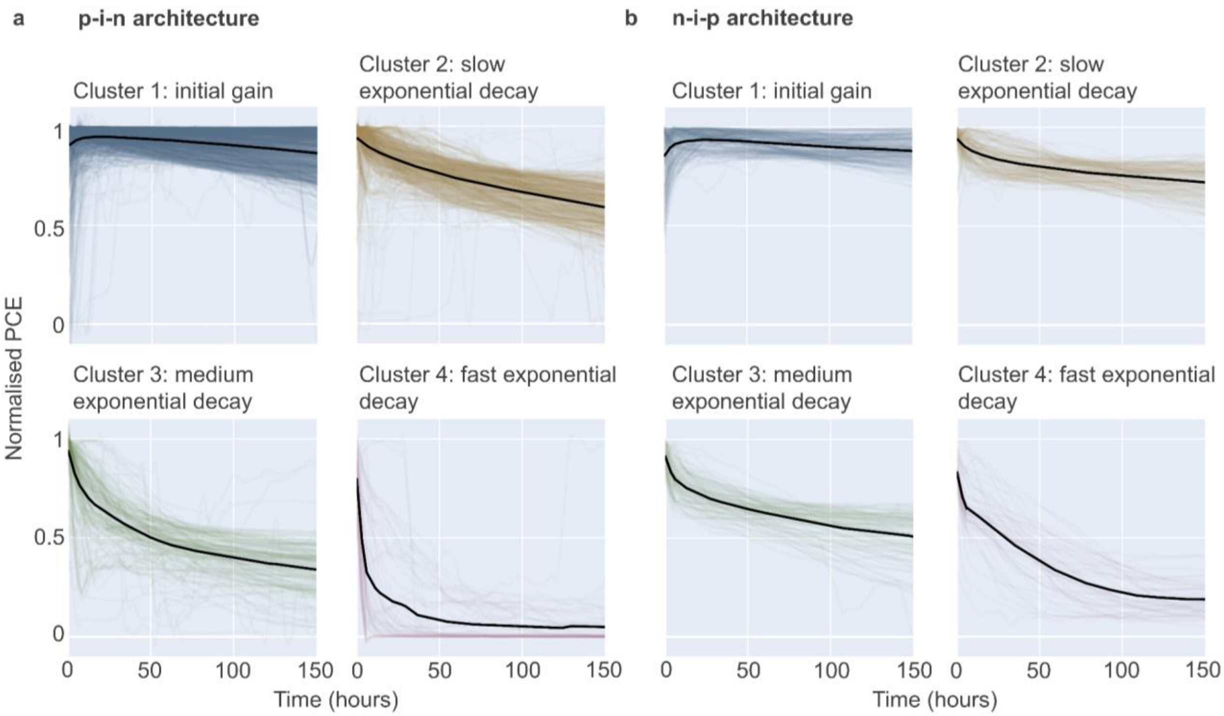

Supplementary Fig. 18. The SOM clusters for **a** *p-i-n* and **b** *n-i-p* devices.

Supplementary Table 5 shows the numbers of devices assigned to a certain cluster in dependency of the architecture. For each architecture, cluster 4 has the smallest share of the devices, while cluster 1 has the highest. However, the percentage for specific clusters and architectures varies slightly, with the exponential decay-type of ageing curve dominating the *n-i-p* architecture (57%, sum of red-shaded), and the initial gain curve dominating the *p-i-n* architecture (58%, blue-shaded). The observed variations in cluster types, influenced by the device architecture, have been substantiated through the implementation of statistical analyses such as the Chi-Squared and Bayesian Contingency Table tests. These findings are consistent with the earlier findings of Saliba et al.[13], which focused on a single-stack configuration. However, it should be emphasised that the association between a specific curve shape and the device architecture cannot be universally generalised.

	Initial gain	Exponential decay		
	**Cluster 1**	**Cluster 2**	**Cluster 3**	**Cluster 4**
***p-i-n* architecture**	1,016 (58%)	503 (29%)	170 (10%)	54 (3%)
***n-i-p* architecture**	214 (43%)	152 (30%)	92 (18%)	44 (9%)

Supplementary Table 5. The breakdown of number of devices based on their architectures and clusters. The blue-shaded box represents the initial gain cluster for *p-i-n* architecture, and the red-shaded box represents the exponential decay clusters for *n-i-p* architecture.

Correlation between Clusters and Testing Conditions

Pearson's correlation coefficient measures linear correlation between two variables, and its value ranges from -1 to 1, with positive value indicates positive correlation and vice versa. **Supplementary Fig. 19** shows that the average temperature during ageing is weakly correlated (|Pearson's correlation coefficient| < 0.3) with all types of clusters. Cluster 3 and 4 in particular have a very low correlation coefficient (0.04 and -0.05) with the average test temperature. Despite cluster 1's (initial gain) low correlation coefficient with average temperature, this cluster is generally observed at lower ageing temperature, based on its correlation coefficient of -0.21. Oppositely, cluster 2 (slow exponential decay) generally appears at higher ageing temperature, based on its correlation coefficient of 0.23. While a correlation coefficient < 0.3 indicates a very weak correlation, the result indicates that the curve shape is also slightly influenced by the temperature. It may be the case that the initial gain period happens much faster at elevated temperatures and the slow exponential decay curve appears as an accelerated version of the initial gain curve, with the curve shape shrank to earlier real time. No correlation between the irradiance (constant at 1000 W/m^2) and clusters is observed and a neglectable correlation between the presence of a UV filter and the clusters is observed.

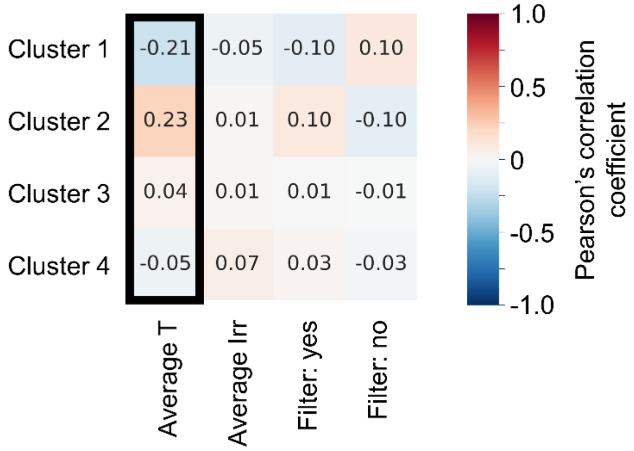

Supplementary Fig. 19. The Pearson's correlation coefficient between the clusters and the degradation conditions (average temperature, average irradiation level, filters). The highlighted box shows the correlation coefficient between average temperature and clusters in particular.

To see how specific average temperature affects the shape of degradation, the dataset is grouped based on the largest average temperature groups during the ageing test, (1) <30°C (1,594 data points) and (2) >80°C (501 data points), as shown in **Supplementary Table 6**. This grouping result supports Pearson's correlation coefficient result. The cells aged at average room temperature (<30°C) mostly have the initial gain shape (61%), while the cells aged at average temperature >80°C mostly have the exponential decay shape (65%).

	Initial gain	Exponential decay		
	**Cluster 1**	**Cluster 2**	**Cluster 3**	**Cluster 4**
**Average T <30°C**	971 (61%)	369 (23%)	171 (11%)	83 (5%)
**Average T >80°C**	174 (35%)	244 (49%)	67 (13%)	16 (3%)

Supplementary Table 6. The breakdown of the number of devices based on their average temperatures. The blue-shaded box represents the initial gain cluster for average temperature <30°C, and the red-shaded box represents the exponential decay clusters for average temperature >80°C.

Data Quality

The analysed dataset has a very high quality in comparison to large perovskite datasets collected from literature. Firstly, the solar cells were fabricated in the same laboratory, and the ageing data was collected in the same ageing system. This potentially reduces various variances compared to literature-mined datasets, where data is compiled from different labs, with different setups and conditions. Secondly, detailed 'features', including device information (materials in each layer) and ageing conditions (temperature, irradiance, spectrum, UV-filter), are collected in this dataset, with no entries missing and with comparable conditions during the test. For example, the ISOS-L-1I protocol[14] allows for MPP or V_{OC} conditions during the test, and in a large literature-mined dataset these may be analysed together, while the different electronic conditions will likely lead to different results of the ageing test[15]. Here, we have exclusively MPP-tracking as the electronic ageing condition. Lastly, we analyse the full MPP-tracks in opposition to an analysis of stability with respect to a single stability metric like e.g. T_{80}, which (a) is sensitive to the curve shape and (b) exposed to errors during the determination of the figure of merit by the analyst.

K-means Clustering

Besides SOM, we also explore the *k*-means clustering method to group the degradation curve shape. *K*-means clustering is a method to group *x* data points into *n* clusters, based on the nearest mean (i.e. cluster centroid) of each data point.[16] **Supplementary Fig. 7** shows the elbow plot of the k-means clustering. **Supplementary Fig. 8** shows the result for *k*-means clustering with *n* = 4, the same as the SOM results. The k-means clustering results generally agree with the SOM results we discussed in the main text.

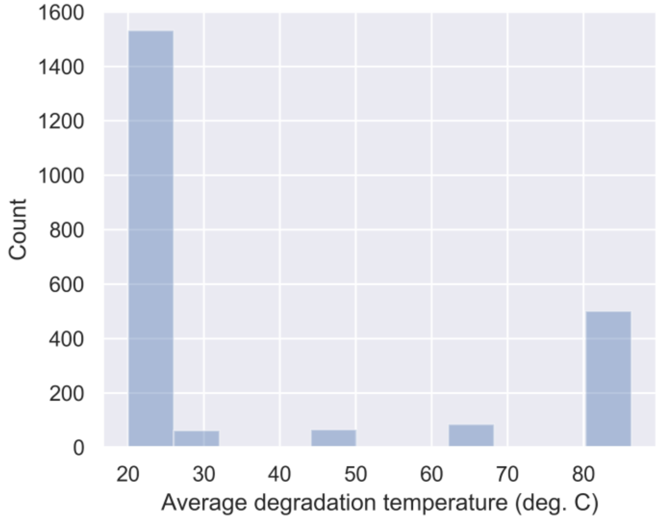

Supplementary Fig. 20. The distribution of ageing temperatures in the dataset.

Supplementary References

1. Eperon, G. E. *et al.* Formamidinium lead trihalide: a broadly tunable perovskite for efficient planar heterojunction solar cells. *Energy Environ. Sci.* **7**, 982–988 (2014).
2. Reference Air Mass 1.5 Spectra. https://www.nrel.gov/grid/solar-resource/spectra-am1.5.html.
3. Marcus Chuang. Shockley-Queisser-limit at master · marcus-cmc/Shockley-Queisser-limit. *GitHub* https://github.com/marcus-cmc/Shockley-Queisser-limit.
4. Saliba, M. *et al.* Cesium-containing triple cation perovskite solar cells: improved stability, reproducibility and high efficiency. *Energy Environ. Sci.* **9**, 1989–1997 (2016).
5. Møller, C. K. Crystal Structure and Photoconductivity of Cæsium Plumbohalides. *Nature* **182**, 1436–1436 (1958).
6. Emery, Q. *et al.* Encapsulation and Outdoor Testing of Perovskite Solar Cells: Comparing Industrially Relevant Process with a Simplified Lab Procedure. *ACS Appl. Mater. Interfaces* **14**, 5159–5167 (2022).
7. Tsarev, S. *et al.* A new polytriarylamine derivative for dopant-free high-efficiency perovskite solar cells. *Sustain. Energy Fuels* **3**, 2627–2632 (2019).
8. Chen, B. *et al.* Grain Engineering for Perovskite/Silicon Monolithic Tandem Solar Cells with Efficiency of 25.4%. *Joule* **3**, 177–190 (2019).
9. Chen, W. *et al.* Surface Reconstruction for Stable Monolithic All-Inorganic Perovskite/Organic Tandem Solar Cells with over 21% Efficiency. *Adv. Funct. Mater.* **32**, 2109321 (2022).
10. Sutton, R. J. *et al.* Bandgap-Tunable Cesium Lead Halide Perovskites with High Thermal Stability for Efficient Solar Cells. *Adv. Energy Mater.* **6**, 1502458 (2016).
11. Yao, Q. *et al.* Dual Sub-Cells Modification Enables High-Efficiency n–i–p Type Monolithic Perovskite/Organic Tandem Solar Cells. *Adv. Funct. Mater.* **33**, 2212599 (2023).
12. Vettigli, G. MiniSom: minimalistic and NumPy-based implementation of the Self Organizing Map. (2018).

13. Saliba, M., Stolterfoht, M., Wolff, C. M., Neher, D. & Abate, A. Measuring Aging Stability of Perovskite Solar Cells. *Joule* **2**, 1019–1024 (2018).
14. Khenkin, M. V. *et al.* Consensus statement for stability assessment and reporting for perovskite photovoltaics based on ISOS procedures. *Nat. Energy* **5**, 35–49 (2020).
15. Domanski, K., Alharbi, E. A., Hagfeldt, A., Grätzel, M. & Tress, W. Systematic investigation of the impact of operation conditions on the degradation behaviour of perovskite solar cells. *Nat. Energy* **3**, 61–67 (2018).
16. Lloyd, S. Least squares quantization in PCM. *IEEE Trans. Inf. Theory* **28**, 129–137 (1982).
